# A Case of the Perinatal Form Hypophosphatasia Caused by a Novel Large Duplication of the *ALPL* Gene and Report of One Year Follow-up with Enzyme Replacement Therapy

**DOI:** 10.4274/jcrpe.galenos.2018.2018.0217

**Published:** 2019-09-03

**Authors:** Bülent Hacıhamdioğlu, Gamze Özgürhan, Catarina Pereira, Emre Tepeli, Gülşen Acar, Serdar Cömert

**Affiliations:** 1 İstinye University Faculty of Medicine, Department of Pediatric Endocrinology, İstanbul, Turkey; 2 University of Health Sciences, Süleymaniye Women Maternity and Child Diseases Training and Research Hospital, Clinic of Child Health and Diseases, İstanbul, Turkey; 3 Centogene AG, Rostock, Germany

**Keywords:** Hypophosphatasia, perinatal form, ALPL gene, duplication, enzyme replacement therapy

## Abstract

Hypophosphatasia (HPP) is a rare disease caused by mutations in the *ALPL* gene encoding tissue-non-specific isoenzyme of alkaline phosphatase (TNSALP). Duplications of the *ALPL* gene account for fewer than 1% of the mutations causing HPP. It has been shown that asfotase alfa enzyme replacement treatment (ERT) mineralizes the skeleton and improves respiratory function and survival in severe forms of HPP. Our patient was a newborn infant evaluated for respiratory failure and generalized hypotonia after birth. Diagnosis of HPP was based on low-serum ALP activity, high concentrations of substrates of the TNSALP and radiologic findings. On day 21 after birth, ERT using asfotase alfa (2 mg/kg three times per week, subcutaneous injection) was started. His respiratory support was gradually reduced and skeletal mineralization improved during treatment. We were able to discharge the patient when he was seven months old. No mutation was detected in the *ALPL* gene by all exon sequencing, and additional analysis was done by quantitative polymerase chain reaction (qPCR). As a result, a novel homozygote duplication encompassing exons 2 to 6 was detected. Early diagnosis and rapid intervention with ERT is life-saving in the severe form of HPP. qPCR can detect duplications if a mutation cannot be detected by sequence analysis in these patients.

**What is already known on this topic?**

Hypophosphatasia (HPP) is caused by mutations in the gene encoding tissue-non-specific isoenzyme of alkaline phosphatase. Missense mutations are the most common type of mutations described for this gene, while duplications are rarely described. It has been shown that enzyme replacement therapy (ERT) mineralizes the skeleton and improves respiratory function and survival in the life-threatening perinatal form of HPP.

**What this study adds?**

We report a novel, large, homozygous duplication encompassing exons 2 to 6 of the ALPL gene. Early diagnosis and rapid intervention with ERT is life-saving in the severe form of HPP.

## Introduction

Hypophosphatasia (HPP) is a rare disease caused by mutations in the gene encoding tissue-non-specific isoenzyme of alkaline phosphatase (TNSALP) (1). It is estimated that the incidence of severe forms of the disease is approximately 1 in 300,000 in Europe and approximately 1 in 435,517 in Turkey (2,3). Patients have been classified traditionally as having perinatal, infantile, childhood, or adult HPP based on symptom severity and presentation age. Currently, specific bone-targeted recombinant enzyme replacement therapy (ERT) (asfotase alfa; STRENSIQ^®^, Alexion Pharmaceuticals, Inc. NASDAQ: ALXN, Boston, Massachusetts, U.S.) is available for HPP patients, and it is suggested for patients with pediatric-onset HPP (1,4).

ALPs are membrane-bound ectoenzymes that hydrolyze monophosphate esters. Human ALP is classified into four types: TNSALP, intestinal, placental-like ALP (PLAP) and germ cell. The expression of TNSALP is widespread, especially in the liver, bone, kidney, neuronal cells and neutrophils. It is expressed on the cell membrane of hypertrophic chondrocytes, osteoblasts and odontoblasts and is also concentrated on the membranes of budding matrix vesicles in these cells. TNSALP is essential for tissue biomineralization (5,6,7).

TNSALP is encoded by an ALP-liver *(ALPL)* gene on chromosome 1p36.12. To date, over 300 different mutations in *ALPL* gene have been identified. Missense mutations are the most common type of mutation. Duplications in this gene have been reported very rarely. Herein, we report a novel duplication in the *ALPL* gene in a patient with the perinatal form of HPP, as well as the patient’s clinical characteristics and a brief report of the results of 12-months follow-up with ERT.

## Case Report

**Figure 1:**
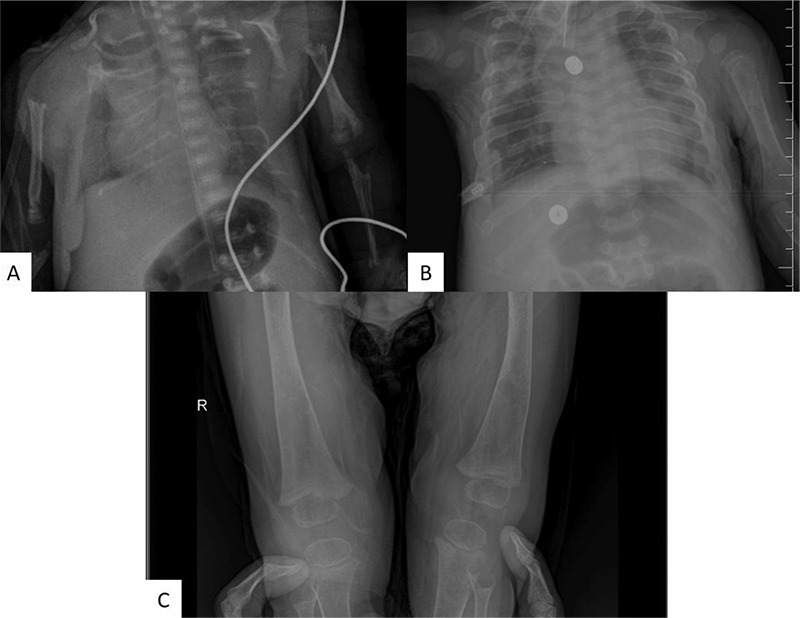
X-ray of the patient; (A) before treatment, (B and C) at 12 months of treatment. Note the general improvement of mineralization and of rachitic changes with asfotase alfa enzyme replacement therapy

The patient was evaluated at birth for respiratory failure and generalized hypotonia. He was born from second cousin consanguineous parents at full-term weighing 3,440 g. The birth length was 50 cm and the head circumference was 35 cm. Diagnosis of HPP was based on low-serum ALP activity, high levels of substrates of TNSALP (see Table 1) and radiologic findings (see Figure 1). The parents were of Turkish origin and healthy. At the time of the assessment, when father’s age was 37 years and mother’s age was 32 years, neither parent had clinical symptoms of HPP.

**Figure 2:**
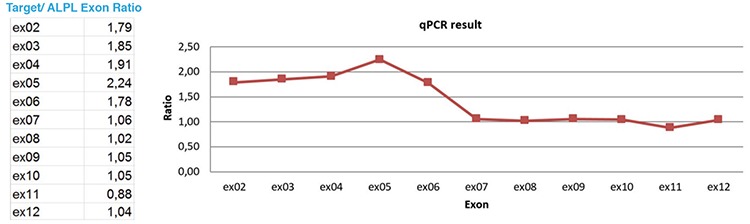
Quantitative polymerase chain reaction (qPCR) assay by using 11 gene-specific amplicons encompassing the coding exons 2 to 12 of the *ALPL* gene. Normalized qPCR ratios are WT (0.70-1.35) and homozygous duplication (4n) (1.75 -2.35) qPCR: quantitative polymerase chain reaction

**Figure 3:**
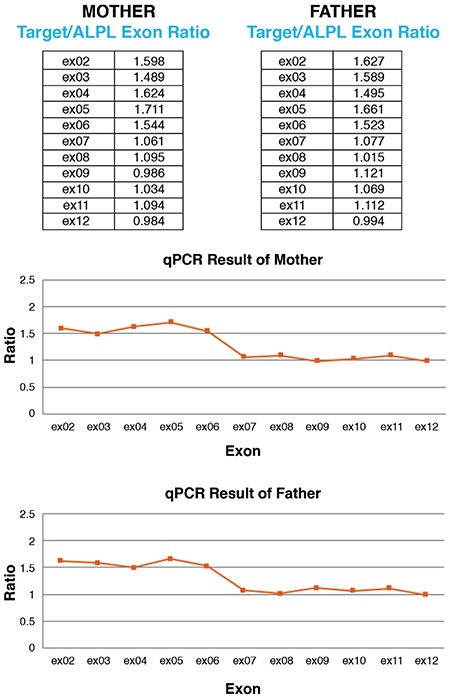
Mother and father heterozygous carriers of the same mutation qPCR: quantitative polymerase chain reaction

No mutation was detected in the *ALPL* gene by full gene sequencing, and thus, we decided to do an additional analysis by quantitative polymerase chain reaction (qPCR). Blood samples were collected from the patient and parents. DNA isolation was performed by a salt precipitation method. The qPCR analysis was performed by LightCycler 480 Software (Roche, Basel, Switzerland), and a relative quantification analysis was performed that compared the target DNA sequence (that of the patient) with a reference DNA sequence (used for normalization of the ratio). Primers were designed for the coding exons 2 to 12 of the gene of interest; 0.5 µL primer forward, 0.5 µL primer reverse, 10 µL SYBR Green I Master (Roche, Basel, Switzerland) and 2 µL DNA were used for reaction mix in a total volume of 20 µL. As a result, a novel homozygous duplication encompassing exons 2 to 6 was detected (Figure 2). This mutation was classified as likely pathogenic (class 2) according to the American College of Medical Genetics (ACMG) and Centogene’s guidelines. Genetic analysis of the parents demonstrated that both were carriers of the same mutation (Figure 3).

Asfotase alfa was kindly provided by Alexion Pharmaceuticals as part of the compassionate use program. On day 21 after birth, ERT using asfotase alfa (2 mg/kg three times per week, subcutaneous injection) was started.

After birth, the patient was intubated and ventilated by Synchronized Intermittent Mandatory Ventilation mode. The inspiratory requirement was gradually reduced during treatment. Due to an ongoing requirement for mechanical ventilation, tracheostomy was performed at the age of six months. The patient was discharged from the hospital at seven months of age. At 12 months he needed ventilation via tracheostomy only during sleep, equivalent to eight hours a day.

Improved mineralization was observed during the treatment (Figure 1). There was no significant hypercalcemia before the treatment and hypocalcemia was not observed during the same period. At age one year, the patient was able to sit up with support, with full head control, but he was not yet able to stand. No objective test, such as the Bayley Scales of Infant and Toddler Development, was performed during the first 12 months to evaluate neurocognitive functions. However we noted some signs of neuromotor development, for example placing a spoon in a cup. We could not evaluate speech function due to the tracheostomy. Seizures were not observed before or during treatment, and there was no sign of craniosynostosis at age 12 months. At this time his weight was 8.0 kg [standard deviation score (SDS) -1.87], his height was 75.0 cm (SDS -0.59), and his head circumference was 46.0 cm (SDS -0.75). He continued to eat normally. Kidney function and renal ultrasound findings were normal. No side effects were observed during the first 12 months of treatment. The patient is currently still on treatment with asfotase alfa, and we hope to share long-term follow-up results in the future.

Written informed consent was obtained from the parents of the patients.

## Discussion

To our knowledge large duplications on the *ALPL* gene have not been reported to date, but minor duplications are not rare (8,9). Herein, we report a novel large homozygote duplication encompassing exons 2 to 6 of the *ALPL* gene. Missense mutations are the most commonly reported type of mutation in this gene (9). This case highlights that, in a patient clinically diagnosed with HPP, duplication or deletion analysis should be performed if a mutation cannot be detected by sequencing.

Recently, it has been shown that ERT with asfotase alfa mineralizes the skeleton and improves respiratory function and survival in the life-threatening perinatal form of HPP (9,10). For patients with a perinatal form of HPP who receive ERT, many of whom had previously died in infancy, survival is the main goal, but not the only goal. Other goals of the treatment are improvement of respiratory status, skeletal mineralization, improvement of growth and physical development, promotion of normal developmental milestones, treatment of craniosynostosis, seizure control and reduced hospitalization requirement (11). We were able to discharge our patient when he was seven months old. His respiratory support was gradually reduced and skeletal mineralization improved during treatment. Asfotase alfa treatment has a good safety profile for children. Common adverse reactions are hypersensitivity reactions, localized lipodystrophy, ectopic calcification of the eye and nephrocalcinosis. Severe hypocalcemia has also been reported (11,12). We monitored our patient according to the current guidelines, and no adverse reactions were observed during the first 12 months (11).

In conclusion, this report describes a child diagnosed with the perinatal form of HPP with a novel large duplication in the *ALPL* gene. Although we were unable to perform cDNA studies/mRNA, this large duplication is very likely to be pathogenic based on ACMG guidelines. Early diagnosis and rapid intervention with ERT is life-saving in the severe form HPP. In patients with HPP duplications can be detected by qPCR, if a mutation cannot be detected by sequence analysis.

## Figures and Tables

**Table 1:**
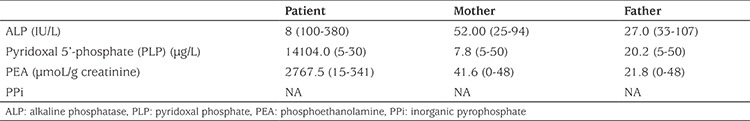
Biochemical findings in the patient and his parents
